# Influence of a Modified versus a Typical Microcycle Periodization on the Weekly External Loads and Match Day Readiness in Elite Academy Soccer Players

**DOI:** 10.5114/jhk/182984

**Published:** 2024-05-17

**Authors:** Tom Douchet, Christos Paizis, Christopher Carling, Nicolas Babault

**Affiliations:** 1 INSERM UMR1093-CAPS, Université de Bourgogne, UFR des Sciences du Sport, Dijon, France; 2 Centre d’Expertise de la Performance, Université de Bourgogne, UFR des Sciences du Sport, Dijon, France; 3 Dijon Football Côte d’Or (DFCO), Dijon, France; 4 Fédération Française de Football (FFF), Paris, France; 5 Laboratory Sport, Expertise and Performance (EA 7370), French Institute of Sport (INSEP), Paris, France

**Keywords:** GPS, youth, training, workload, performance

## Abstract

A typical weekly periodization strategy in soccer positions the most demanding sessions four and three days before the match-day (MD−4/MD−3). However, a modified periodization strategy could intersperse the two most demanding sessions with a low-load training session. This study aimed to investigate the impact of these periodizations on external loads and MD readiness. Twenty academy players were tested. The modified weekly periodization strategy included a low-load training session between the two most demanding sessions, while the typical periodization implemented them consecutively. Players wore a GPS and rated their perceived exertion (RPE) during the most demanding sessions. Players were also tested using a CMJ, a 20-m sprint, the Illinois agility test (IAT), and the Hooper questionnaire on MD−4 to obtain baseline values (CONTROL) and on the match-day (TEST). CONTROL values were similar during experimental weeks. During the second most demanding session, players covered greater distances for the modified versus the typical periodization in the 20–25 km/h (306.3 ± 117.1 m vs. 223.5 ± 92.2 m, p < 0.05) and >25 km/h speed zones (89.5 ± 44.8 m vs. 67.2 ± 44.5 m, p < 0.05). On the MD, CMJ, 20-m sprint, and IAT performances were similar between both periodizations. However, 10-m time (1.89 ± 0.10 s vs. 1.92 ± 0.09 s, p < 0.05) and the Hooper index score (7.90 ± 2.14 a.u. vs. 9.50 ± 3.44 a.u., p < 0.05) were significantly lower during the modified periodization strategy. A decreased training load session positioned between the most demanding sessions may be of interest in elite academy soccer players as it can lead to increased weekly external loads and readiness on the MD.

## Introduction

In soccer, the periodization strategies adopted by elite practitioners aim to ensure optimal recovery following match-play and develop or at least maintain physical qualities for the next match throughout the competitive season. As such, practitioners are constantly trying to optimize their microcycle (i.e., weekly) periodization strategy. However, scientific evidence and rationale for weekly periodization strategies are scarce. A recent study demonstrated that in elite French soccer academies, almost all soccer teams implemented similar microcycle periodization strategies (Douchet et al., 2023). Players are typically exposed to light training loads (volume and intensity) during the two days following the match (match-day+1 [MD+1] and +2 [MD+2]). The load is increased during the next two days to develop or maintain physical qualities. On MD−2 and MD−1, a progressive decrease in training loads is reported for tapering purposes (Douchet et al., 2023). Although some minor differences exist (for instance with a day off on MD+1 or MD+2) (Buchheit et al., 2023), this typical periodization is generally observed in professional soccer settings (Los Arcos et al., 2017; Martín-García et al., 2018; Oliva-Lozano et al., 2022). However, professional and academy youth teams do not necessarily share the same objectives during a competitive microcycle. One can reasonably assume that practitioners in professional settings mainly focus upon preparing their players to achieve competitive results. In contrast, academy policies might emphasize player development over competitive results (Douchet et al., 2023). As such, the similar microcycle periodization strategies (Dimitriadis et al., 2022; Douchet et al., 2023; Lyakh et al., 2014; Martín-García et al., 2018; Plakias 2023) observed in academy and professional settings might seem surprising. Indeed, greater weekly training loads for physical development purposes would arguably be expected in academies.

In soccer, an increase in training loads for physical conditioning purposes can be achieved using different strategies such as increasing the number of sessions and/or their duration (Anderson et al., 2016). However, a combination of higher training loads and longer duration have been shown to deleteriously impact physical performance on the following day (Douchet et al., 2022). Therefore, the adverse effects of such increases might negatively impact the effectiveness of any subsequent training session and their expected effects on physical development. A potential means to increase training loads without any detrimental effect on physical development could involve modification of the typical weekly periodization strategy. Currently, a typical soccer periodization strategy positions the two most physically demanding sessions consecutively on MD−4 and MD−3 (Douchet et al., 2023). Interposing a low-load training session between the two heaviest sessions could arguably favor recovery, prevent or reduce any deterioration in performance, and even potentially allow players to perform greater running loads during the second most demanding training session. This modified periodization strategy (i.e., most demanding sessions on MD−4 and MD−2 with a low-load session in-between on MD−3) has notably been used in rugby union with positive chronic effects on different fitness characteristics (Dubois et al., 2020).

Although a change in the periodization strategy could help increase the training load without alteration of the training content, it might negatively impact readiness to play on the MD. Indeed, the modified periodization would delay and shorten the weekly tapering phase, which typically starts on MD−2 (Douchet et al., 2023). Increasing the training load on MD−2 with intensive training sessions (such as high-speed running) could impair the neuromuscular system up to 48 h post-training (Howatson and Adi, 2009). Moreover, such increases on MD−2 have been shown to decrease the winning probability for the following match in senior players (Oliveira et al., 2020). However, no information exists on the effect of such a modified periodization strategy on MD readiness in elite soccer academy players.

The present study aimed to examine the effects of a modified weekly periodization (with a low-load training session positioned between the two most demanding training sessions) compared to a typical periodization strategy (the two most demanding training sessions performed consecutively) in elite academy soccer players on measures of external training loads and readiness for the subsequent match. We hypothesized that the modified weekly periodization could enable soccer players to produce greater external loads without compromising readiness on the match day. Readiness to play was considered as a combination of physical fitness and a well-being state.

## Methods

### Experimental Approach to the Problem

**Figure 1:**
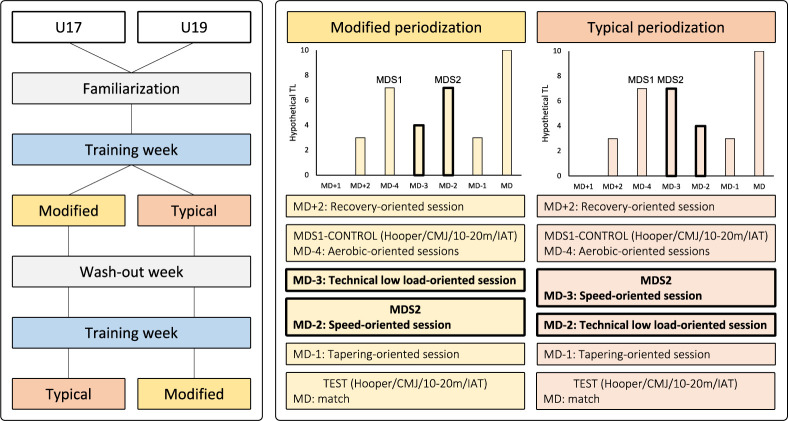
Study flowchart. The two “Training weeks” were of identical contents and served to standardize the physical level before the two experimental weeks. Modified days between the typical and the modified periodization (MD−3 and MD−2) are shown in bold. MDS: Most demanding session; MD: Match-day; CMJ: Countermovement Jump; IAT: Illinois agility test; TL: Training load.

This study used a randomized cross-over design to examine the effects of a modified in-season weekly periodization strategy, compared to a typical strategy, on the training loads performed during the most physically demanding sessions, and subsequent MD readiness (Figure 1). Two experimental weeks were tested. During the typical experimental week, the two most demanding training sessions were performed consecutively on MD−4 and MD−3. During the modified experimental week, a low-load training session interspersed (i.e., on MD−3) the most demanding sessions (performed on MD−4 and MD−2). The remaining sessions of the week were unchanged (Table 1). On the MD, players played a 90-min training match where all players had at least 60-min playing time. Players were monitored on MD−4 to obtain control values for tests of readiness (a countermovement jump [CMJ], a 20-m sprint, the Illinois Agility Test [IAT], the Hooper questionnaire, a training workload using the global positioning system [GPS], and the rate of perceived exertion [RPE]). Players were monitored during the second most demanding session (MD−3 and MD−2 for the typical and modified periodization, respectively) with the GPS and the RPE to evaluate external and internal training workloads, respectively. Players were tested on the MD using a CMJ, a 20-m sprint, the IAT, and the Hooper questionnaire to determine their readiness for the match day. T-tests were used to evaluate possible statistical differences between both types of periodization strategies.

**Table 1:** Organization of the two experimental weeks.

	Modified periodization		Typical periodization	
	AM	PM	AM	PM
**MD+1**	Rest	Rest	Rest	Rest
**MD+2**	Rest	Upper body strength session + Active recovery / prophylactic work (60 min)	Rest	Upper body strength session + Active recovery / prophylactic work (60 min)
**MD-4**	CONTROL 12 min 30"/30" maximal aerobic speed intermittent work + Aerobic oriented session – small- and medium-sided games (90 min)	Positional specific session – technical drills (passing, dribbling, finishing) (60 min)	CONTROL 12 min 30"/30" maximal aerobic speed intermittent work + Aerobic oriented session – small- and medium-sided games (90 min)	Positional specific session – technical drills (passing, dribbling, finishing) (60 min)
**MD-3**	**Technical low-load drills (rondo – football tennis) (75 min)**	**Rest**	**4 x 30 m sprint work + Speed oriented session – large-sided games (90 min)**	**Lower body strength session (squat – deadlift – lunges) (60 min)**
**MD-2**	**4 x 30 m sprint work + Speed oriented session – large-sided games (90 min)**	**Lower body strength session (squat – deadlift – lunges) (60 min)**	**Technical low-load drills (rondo – football tennis) (75 min)**	**Rest**
**MD-1**	Rest	Reactivity – SSG (60 min)	Rest	Reactivity – SSG (60 min)
**MD**	TEST + Match (90 min)	Rest	TEST + Match (90 min)	Rest

Modified days between the typical and modified periodization (MD-3 and MD-2) are shown in bold.

### Participants

A total of twenty-eight elite academy soccer players (14 U17 players: age = 16.1 ± 0.5 years, body height = 170.3 ± 5.7 cm, body mass = 63.4 ± 3.6 kg and 14 U19 players: age = 18.3 ± 0.4 years, body height = 172.4 ± 6.5 cm, body mass = 68.1 ± 6.7 kg) belonging to the French professional Ligue 2 soccer club of Dijon Football Côte d’Or were recruited. According to a previous paper, players were classified as highly-trained athletes (Tier 3) (McKay et al., 2022). The study design was first presented to all the technical staff of this soccer club. After the agreement of most coaches, recruitment was then conducted while first explaining the protocol to U19 players, then U17 players. Goalkeepers were not included due to the different nature of their activity. Players not completing all training sessions were also omitted. Players were instructed to maintain their habitual daily food and water intake. All the players were notified of the research protocol, benefits, and risks before providing written informed consent in accordance with the Declaration of Helsinki. Approval of the study was obtained by the STAPS committee (approval code: IRB00012476-2021-17-06-114; approval date: 10 October 2020). The sample size was calculated a priori using G*Power (version 3.1.9.6, free software available at https://www.psychologie.hhu.de/arbeitsgruppen/allgemeine-psychologie-und-arbeitspsychologie/gpower.html) using the following values: effect size of 0.35, power of 0.8, probability error of 0.05. A sample size of 19 volunteers was indicated. Of the 28 volunteers recruited, 20 players (11 U17 players and 9 U19 players) were retained for statistical analyses. The eight other players were excluded owing to injuries sustained. Four of these players were excluded before any data collection (two before randomization and two before the first experimental week). The four others were excluded before the second experimental week. The values obtained from these four volunteers (first experimental week) were not retained for analyses because the a priori sample size was attained.

### Procedures

Players were tested during the 2020–2021 season over six consecutive weeks, following the winter break, between the 33^rd^ and the 38^th^ week of the season. Two experimental weeks were evaluated with each preceded by a standardized training week. The experimental weeks were presented in random order pre-determined using www.randomizer.org. The two blocks (standardized week + experimental week) were interspersed by a standardized wash-out week (Figure 1) to ensure similar baseline values (Hecksteden et al., 2018; Kritikos et al., 2021). To limit possible confounding effects, standardized training weeks were strictly identical. During the experimental weeks, training content, duration and intensity were identical. For the typical periodization strategy, two heavy-load sessions were implemented on MD−4 and MD−3 and were defined as the most demanding session 1 and the most demanding session 2, respectively. These were followed by a low-load session on MD−2 (Figure 1). During the modified periodization approach, the contents of the training sessions on MD−3 and MD−2 were inverted. As such, during the modified periodization, the two most demanding sessions were conducted on MD−4 and MD−2 and were interspersed by a low-load session on MD−3 (Figure 1).

Dissociated physical work was executed during the two most demanding sessions. Players performed a similar aerobic-oriented session on the first most demanding session (performed on MD−4 for both experimental conditions) (Table 1). This session included 12 min of 30"/30" (30-s effort / 30-s passive rest) maximal aerobic speed intermittent work followed by 20-min (5 x 4 min, 2-min rest, 5 vs. 5 with 125 m^2^ per player) small- and 20-min (4 x 5 min, 2-min rest, 5 vs. 5 with 175 m^2^ per player) medium-sided games. Players performed a similar speed-oriented session during the second most demanding session (on MD−3 for the typical periodization and MD−2 for the modified periodization) (Table 1). This session involved a series of 4 x 30 m sprints followed by 40-min (4 x 10 min, 3 min rest, 10 vs. 10) large-sided games. All field training sessions took place on an artificial soccer pitch.

Training workloads were measured during the two most demanding sessions. External and internal loads during the first most demanding session (on MD−4) served as CONTROL values. During the second most demanding session (typical periodization: MD−3; modified periodization: MD−2), the external and internal training loads were used to compare the typical and modified periodization strategy.

Training load monitoring was performed using a 10-Hz Fieldwiz GPS (Fieldwiz, Liège, Belgique), integrating a 100-Hz triaxial accelerometer microsensor for every field-based training session and match. This tracking device has been shown to display low bias and a good coefficient of variation for total distance covered and peak speed, indicating sufficient reliability to track team-sport load variables (Willmott et al., 2019). The device was worn between the scapulae using a specifically designed vest. As recommended by the manufacturer, all devices were activated 15 min before data collection. GPS devices were distributed to players 10 min before the training session onset. Players systematically wore the same device to avoid inter-unit variability. GPS devices were turned off as soon as the training session or the match was terminated. Data were downloaded and analyzed immediately after each match and training session using the manufacturer’s propriety software package (Fieldwizz, ASI, Lausanne, Switzerland). The external load was monitored using the following variables: total distance (TD) expressed as the absolute distance covered by players and in meters (m), distances at specific speeds (in m) including low-speed distance (LSD; [0–15] km·h^−1^), moderate-speed distance (MSD; [15–20] km·h^−1^), high-speed distance (HSD; [20–25] km·h^−1^), and sprint distance (SPR; > 25 km·h^−1^), and the total number of accelerations (ACC; > 3 m·s^−2^) and decelerations (DEC; < −3 m·s^−2^). The same thresholds were used for all players (Coutinho et al., 2015; Wrigley et al., 2012). Following these experimental training sessions, players reported their RPE using a Borg CR-10 scale (Douchet et al., 2022) on their individual cellphones between 15 to 30 min after the end of the training session (Douchet et al., 2022).

Physical performance was assessed during MD−4 and the MD. On MD−4, values served as CONTROL to ensure that players had similar physical levels before the first most demanding training session of the two experimental weeks. Tests were conducted in the morning of the first most demanding session. On the MD, the testing procedure (here called TEST) was used to compare readiness at the end of the typical and the modified periodization. The testing procedure was chosen in accordance with that used in a previous study (Douchet et al., 2022). Tests included a CMJ, a 20-m sprint, the IAT, and the Hooper questionnaire. Players first had to answer the Hooper Questionnaire to subjectively determine their readiness according to ratings of sleep quality of the preceding night, fatigue, stress, and delayed onset muscle soreness (DOMS) (Hooper and Mackinnon, 1995). Each response was rated on a seven-point Likert scale, with responses ranging from 1 to 7 corresponding to “very, very good” to “very, very bad” for sleep and to “very, very low” to “very, very high” for fatigue, stress, and DOMS. The Hooper Index (HI) summated the four ratings (Hooper and Mackinnon, 1995). Ratings were completed on players’ individual cellphones, 30 min before the session, to limit any potential influence from teammates.

Participants then performed a 15-min standardized FIFA 11+ warm-up (O’Brien et al., 2017) followed by physical tests systematically completed in the following order: a CMJ, a 20-m sprint, and the IAT. Players had two trials for each test, interspersed by 2 min of passive recovery. The different tests were also separated by 2-min passive recovery. The best values were retained for statistical analysis (Brini et al., 2022). Players were familiarized with the testing procedure one week before the study onset (Figure 1). Regarding the CMJ, players had to jump as high as possible, beginning in a standing position, then flexing the knees until 90°, and extending the knees to jump in a continuous movement (Malone et al., 2015). They were asked to keep their arms on their hips from standing until landing. Performance was measured using a photocell jump system (Optojump, Microgate, Bolzano, Italy) sampling at 1000 Hz, with jump height (cm) subsequently calculated by proprietary software (Optojump, Version 1.3.20.0, Microgate, Bolzano, Italy). During the 20-m sprint, 10-m, and 20-m sprint times were measured using three pairs of photoelectric timing gates (Witty system, Microgate, Bolzano, Italy). Players started from a standing position as close to the timing gates as possible without triggering the cells. The IAT (Amiri-Khorasani et al., 2010) was performed after the 20-m sprint test to assess soccer-specific speed abilities. The IAT time was measured using timing gates (Witty system, Microgate, Bolzano, Italy).

### Statistical Analyses

Statistical analyses were conducted using JASP (version 0.14, JASP Team 2020, University of Amsterdam, available free at https://jasp-stats.org/download/, accessed on 20 November 2021). Results are presented as mean values (± standard deviation, SD) and mean difference (MD) with 95% confidence intervals. Normality was tested and confirmed for all variables using the Shapiro-Wilk test, and parametric statistics were subsequently used to analyze all indicators. Paired student *t*-tests were used to analyze differences between the two experimental weeks. Statistical significance was set at *p* < 0.05. Subsequently, qualitative descriptors of standardized effects were used for pairwise comparison with Cohen's *d* with 95% confidence intervals. Their interpretation was based on the following criteria: < 0.2 = trivial effect, 0.2 to 0.6 = small effect, 0.6 to 1.2 = moderate effect, 1.2 to 2.0 = large effect and > 2.0 = very large effect. Since U17 and U19 players were considered, the age-group could be a confounding factor. This effect was tested using Student’s *t*-test for physical performance and using two-way analyses of variances while considering the experimental weeks and age-group as main factors. Because no age-group effect was obtained, and for simplicity, age-group effects were not presented in the subsequent parts. Similarly, no effect of the order of the sequences was observed and was therefore not presented.

## Results

### Training Workload

The field session performances are shown in Table 2. Analysis of the most demanding session 1 for the CONTROL condition demonstrated similar values for both experimental weeks: TD (*p* = 0.580; MD = 76.33 (−359.95–207.29); *d* = 0.12 (−0.56–0.32), trivial); LSD (*p* = 0.728; MD = 34.64 (−239.76–170.48); *d* = 0.07 (−0.52–0.36), trivial); MSD (*p* = 0.588; MD = 32.68 (−156.79–91.42); *d* = 0.12 (−0.56–0.32), trivial); HSD (*p* = 0.682; MD = 26.32 (−106.22–158.87); *d* = 0.09 (−0.35–0.53), trivial); SPR (*p* = 0.157; MD = −36.36 (−87.98–15.27); *d* = −0.33 (−0.78–0.12), small); ACC (*p* = 0.924; MD = −0.40 (−9.05–8.25); *d* = 0.02 (−0.46–0.42), trivial); DEC (*p* = 0.825; MD = 0.90 (−7.50–9.30); *d* = 0.05 (−0.39–0.49), trivial) and RPE (*p* = 0.359; MD = 0.20 (−0.24–0.64); *d* = 0.21 (−0.24–0.65), small).

**Table 2:** Typical and modified periodization external and internal training loads during the most demanding sessions 1 and 2.

	Most demanding session 1		Most demanding session 2	
	Typical	Modified	Typical	Modified
TD (m)	6689.2 ± 429.9	6765.6 ± 629.7	6033.5 ± 678.6	5919.5 ± 525.7
LSD (m)	4570.5 ± 348.5	4605.1 ± 411.6	5246.1 ± 686.7	4930.0 ± 461.3
MSD (m)	1390.9 ± 172.4	1423.57 ± 207.4	563.9 ± 206.1	592.2 ± 153.8
HSD (m)	683.3 ± 302.2	657.0 ± 291.7	223.5 ± 92.3	306.4 ± 117.1*
SPR (m)	43.9 ± 34.9	80.3 ± 98.0	67.2 ± 44.6	89.6 ± 44.9*
ACC (n)	79.1 ± 14.2	79.5 ± 16.7	87.6 ± 24.2	87.3 ± 21.5
DEC (n)	54.9 ± 13.1	54.0 ± 16.7	66.8 ± 21.0	61.4 ± 18.6
RPE (a.u.)	7.2 ± 0.9	7.0 ± 0.9	5.9 ± 1.3	6.6 ± 1.1

Values are presented as means ± SD. Most demanding session 1 served as CONTROL. Significantly greater performance between the typical and the modified periodization are shown (* *p* < 0.05). TD: Total distance; LSD: Low speed running ([0–15] km·h^−1^); MSD: Moderate speed distance ([15–20] km·h^−1^); HSD: High speed distance ([20–25] km·h^−1^); SPR: Sprint (> 25 km·h^−1^); ACC: Acceleration (> 3 m·s^−2^); DEC: Deceleration (< −3 m·s^−2^); RPE: Rate of perceived exertion.

For the most demanding session 2, most indicators did not differ significantly between the typical and the modified periodization strategy: TD (*p* = 0.552; MD = 113.94 (−279.59−507.48); *d* = 0.13 (−0.31−0.57), trivial); LSD (*p* = 0.140; MD = 316.09 (−113.68−745.86); *d* = 0.34 (−0.11−0.79), small); MSD (*p* = 0.568; MD = −28.34 (−130.34−73.66); *d* = 0.13 (−0.57−0.31), trivial); ACC (*p* = 0.919; MD = 0.35 (−6.75−7.45); *d* = 0.02 (−0.42−0.46), trivial); DEC (*p* = 0.134; MD = 5.40 (−1.83−12.63); *d* = 0.35 (−0.11−0.79), small) and RPE (*p* = 0.110; MD = −0.35 (−0.79−0.09); *d* = 0.37 (−0.82−0.08), small). In contrast, HSD (*p* = 0.025; MD = −82.84 (−153.90−11.77); *d* = −0.54 (−1.01−0.07), small) and SPR (*p* = 0.015; MD = −22.35 (−39.83−4.87); *d* = −0.59 (−1.07−0.11), small) were significantly greater during the modified versus the typical periodization strategy.

### Testing Procedure

Values for the physical tests and the Hooper questionnaire during CONTROL (Table 3) were not significantly different between the two monitored weeks: CMJ (*p* = 0.268; MD = 0.69 (−0.58−1.96); *d* = 0.25 (−0.13−0.39), small); 10 m (*p* = 0.359; MD = −0.02 (−0.05−0.02); *d* = 0.21 (−0.65−0.24), small); 20 m (*p* = 0.937; MD = 0.00 (−0.04−0.04); *d* = 0.02 (−0.42−0.46), small); IAT (*p* = 0.342; MD = −0.17 (−0.54−0.20); *d* = 0.02 (−0.66−0.23), small); Fatigue (*p* = 0.625; MD = −0.15 (−0.78−0.48); *d* = 0.11 (−0.55−0.33), trivial); Sleep (*p* = 0.186; MD = −0.45 (−1.14−0.24); *d* = 0.20 (−0.75−0.15), small); Stress (*p* = 0.577; MD = 0.05 (−0.13−0.23); *d* = 0.13 (−0.31−0.56), trivial); DOMS (*p* = 0.825; MD = 0.10 (−0.11−0.15); *d* = 0.050 (−0.68−0.77), trivial); HI (*p* = 0.060; MD = −1.40 (−2.87−0.07); *d* = 0.44 (−0.90−0.02), small).

**Table 3:** Values for physical tests and the Hooper questionnaire during the typical and modified periodization strategies for the most demanding session 1 (CONTROL: MD−4).

	Typical periodization	Modified periodization
CMJ height (cm)	41.68 ± 4.94	41.57 ± 4.19
10-m sprint (s)	1.90 ± 0.09	1.90 ± 0.09
20-m sprint (s)	3.20 ± 0.15	3.19 ± 0.13
IAT (s)	15.42 ± 0.59	15.45 ± 0.45
Fatigue (au)	2.55 ± 1.02	2.55 ± 1.20
Sleep (au)	2.20 ± 1.03	2.50 ± 1.20
Stress (au)	1.35 ± 0.73	1.70 ± 1.49
DOMS (au)	2.00 ± 0.84	2.00 ± 0.95
Hooper Index (au)	8.10 ± 2.28	8.75 ± 3.13

Values are means ± SD. CMJ: Counter Movement Jump; IAT: Illinois Agility Test; DOMS: Delayed Onset Muscle Soreness; au: arbitrary unit.

**Figure 2:**
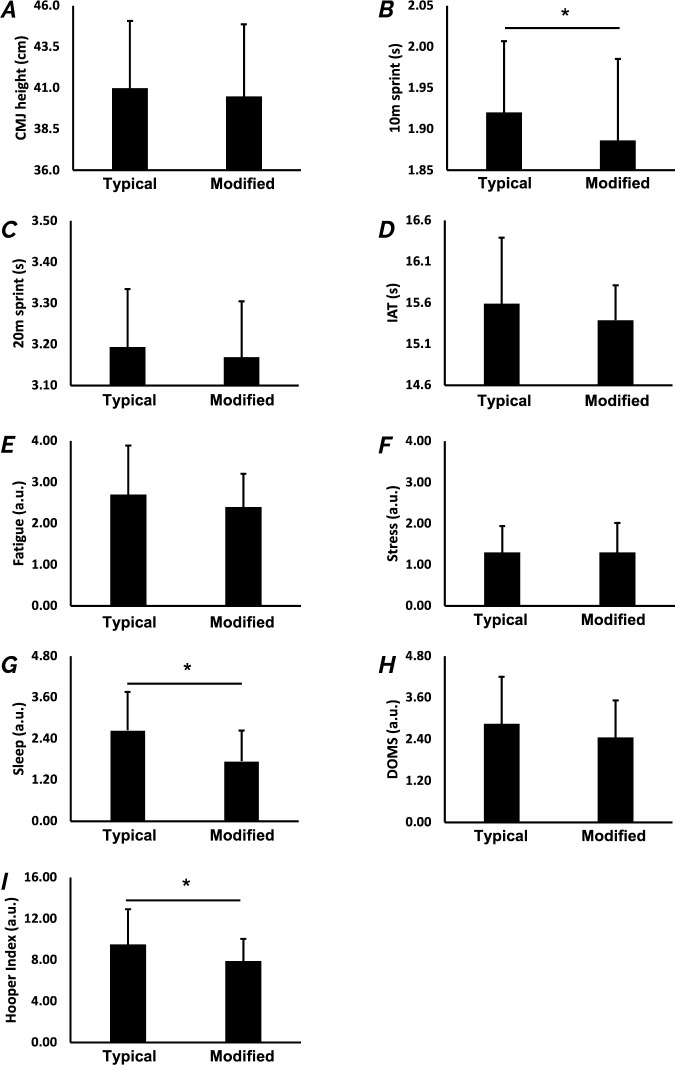
Physical and Hooper questionnaire results for TEST on the MD. Significantly greater performance for physical tests and subjective readiness level for the Hooper questionnaire are observed (* *p* < 0.05). A: Countermovement jump height; B: 10-m sprint time; C: 20-m sprint time; D: Illinois Agility Test time; E: Fatigue; F: Stress; G: Sleep; H: DOMS (Delayed Onset Muscle Soreness); I: Hooper Index.

Under the TEST condition, values for the CMJ (*p* = 0.499; MD = 0.50 (−1.02−2.02); *d* = 0.15 (−0.29−0.59), trivial); 20 m (*p* = 0.138; MD = 0.02 (−0.01−0.06); *d* = 0.34 (−0.11−0.80), small); IAT (*p* = 0.186; MD = 0.20 (−0.10−0.50); *d* = 0.30 (−0.14−0.75), small); Fatigue (*p* = 0.249; MD = 0.30 (−0.23−0.83); *d* = 0.27 (−0.18−0.71), small), Stress (*p* = 1.000; MD = 0.00 (−0.21−0.21); *d* = 0.00 (−0.43−0.43), trivial), and DOMS (*p* = 0.134; MD = 0.40 (−0.13−0.3); *d* = 0.3 (−0.106−0.80), small) were not significantly different (Figure 2). In contrast, 10-m time was significantly lower during the modified periodization as compared to the typical periodization (*p* = 0.024; MD = 0.03 (0.01−0.06); *d* = 0.55 (0.07−1.02), small). Values for sleep (*p* = 0.014; MD = 0.90 (0.20−1.60); *d* = 0.60 (0.12−1.08), moderate) and HI (*p* = 0.016; MD = 1.60 (0.33−2.86); *d* = 0.59 (0.11−1.06), small) were also lower during the modified weeks as compared to the typical periodization (Figure 2).

## Discussion

The present study investigated the effects of a modified weekly periodization strategy compared to a typical periodization in elite academy soccer players on measures of external training loads and subsequent readiness for the next match. The modified weekly periodization was interspersed with a low-load training session included between the two most demanding training sessions, while these two sessions were consecutive during the typical weekly periodization. Results revealed that the modified periodization led to players performing greater external loads at high intensities during the second most demanding session. Furthermore, the modified periodization strategy did not appear detrimental to subsequent match performance since the readiness level on the MD increased.

Here, it is noteworthy that players were able to perform greater distances at high-speed and sprint during the speed-oriented session in the most demanding session 2 of the modified periodization as compared to the typical strategy. This result suggests that the low-load session placed on MD−3 during the modified periodization was beneficial in helping youth players to recover from the likely fatigue obtained following the most-demanding session 1. Indeed, during the most demanding session 1, practitioners sought aerobic development through small- and medium-sided games. These drills notably require heavy demands related to changes in movement direction leading to greater ACC and DEC demands per min than competition (Sarmento et al., 2018). Authors have previously shown that an elevation in the number of ACCs and DECs led to increases in indicators of muscle damage such as plasma creatine kinase and fatigue (Douchet et al., 2021; Young et al., 2012). Following exercise-induced muscle damage, plasma creatine kinase and DOMS are significantly elevated 24 to 48 h post training intervention (Twist and Eston, 2005). These elevations can lead to similar decreases in sprint performance 24 h and 48 h post-exercise (Twist and Eston, 2005). However, research has shown that implementing an active rather than a passive recovery strategy immediately after an intense soccer training session promoted significantly decreased muscle damage 48h post training intervention (Darani et al., 2018). Therefore, we can hypothesize that the low-load session placed between the two most-demanding training sessions during the modified periodization might have acted as active recovery and as such, was beneficial for subsequent training by alleviating fatigue. Moreover, this periodization strategy could also be of interest to increase high velocity external loading, which is shown to be significantly lower in youth players compared with professionals throughout the week (Houtmeyers et al., 2020). These elevated weekly external loads are important to soccer performance (Di Salvo et al., 2009). They could help prepare players to cope with the ever-increasing high intensity competitive demands (Lago-Peñas et al., 2022; Nassis et al., 2020), and bridge the gap with professional standards (Houtmeyers et al., 2020).

Previous studies have highlighted that a gradual reduction in the training load (tapering) across the final two days leading to competition plays a key role in the periodization strategy to increase players’ readiness (Kelly et al., 2020; Los Arcos et al., 2017). It is noteworthy that the present results demonstrated that the players’ readiness level on the MD, as attested by improved scores in the Hooper questionnaire and physical tests, improved following the modified periodization microcycle. This finding might be explained by the recovery on the day interspersing the two most-demanding training sessions. Without sufficient recovery, successively conducting the two most-demanding sessions (typical periodization strategy) could lead to fatigue accumulation. Indeed, a previous study showed that greater training loads were correlated with a decrease in subjective recovery indices in academy players (Noon et al., 2018). To counteract fatigue accumulation, practitioners usually commence weekly tapering on MD−2 (Douchet et al., 2023). Interestingly, the present results suggest that tapering can be shortened, thereby enabling increased emphasis and positive effects on physical development. Indeed, in addition to the increased training load during the second of the most demanding sessions, the low-load session during the modified periodization strategy on MD−3 positively affected the readiness level on the MD. This conclusion was further highlighted by improved 10-m sprint times on the MD following the modified periodization. An alternative periodization strategy where a highly demanding training session is programmed closer to the match-day is thus feasible when sufficient recovery is scheduled within the microcycle. Given all this information, the modified periodization could be generalized and applied in different contexts. Practical applications by coaches and conditioning trainers could be interesting for players in elite academies to improve external loads in the second most demanding session and increase match day readiness. In amateur soccer players where there are fewer training sessions it is a way to improve physical capacities. In professional teams, the modified periodization in addition to improving the external load in the second most demanding session and increasing match day readiness, could also modify the monotony of training content.

Although this study is new to the field of periodization of soccer microcycles in elite soccer academy players, it nevertheless has certain limitations. The fact that these results are based on a single team in a single season makes them difficult to generalize. Other contextual factors (e.g., coach, weather, etc.) also influence the results obtained. Indeed, training periodization is a complex process depending on numerous factors such as the player’s performance level, inter-individual history, the period during the pre- and in-season, and individual responses to training stimulus. Indeed, players may respond differently to these varying periodization models. In addition, the results of our study analyzed the average responses across the group of players. As a result, some individuals may have responded differently to these periodizations. As such, the present experimental design should be replicated over a longer period using a larger sample size and in different training contexts, but also using a day-by-day readiness monitoring to depict how players respond throughout the week. For instance, additional professional teams combined with different training contents and periodization strategies should be considered, taking into account the individual responses. However, this modified periodization strategy (i.e., most demanding sessions on MD−4 and MD−2 with a low-load session in-between on MD−3) has notably been used in rugby union with positive chronic effects on different fitness characteristics (Dubois et al., 2020).

## Conclusions

The results of the present study demonstrated that a modified periodization (most demanding sessions interspersed with a low-load session) led to greater HSD and SPR distances covered in the speed-specific training session. These increased distances could have possible advantages for the development of physical qualities and notably aid development of youth soccer players’ performance during official competitions. Furthermore, delaying the weekly tapering to MD−1 was not detrimental for players’ readiness levels on the MD. On the contrary, the modified periodization slightly improved some readiness scores for the subsequent competitive game.
